# Organic thermally activated delayed fluorescence material with strained benzoguanidine donor

**DOI:** 10.3762/bjoc.19.95

**Published:** 2023-09-07

**Authors:** Alexander C Brannan, Elvie F P Beaumont, Nguyen Le Phuoc, George F S Whitehead, Mikko Linnolahti, Alexander S Romanov

**Affiliations:** 1 Department of Chemistry, The University of Manchester, Manchester, United Kingdom; 2 Department of Chemistry, University of Eastern Finland, Joensuu, Finland

**Keywords:** guanidine, organic, photoluminescence, TADF, yellow

## Abstract

Organic thermally activated delayed fluorescence (TADF) materials have been widely investigated due to their impressive electronic properties and applied potential for the third generation of organic light-emitting diodes (OLED). We present organic TADF material (**4BGIPN**) based on the strained benzoguanidine donor and compare it with the benchmark carbazole-based material (**4CzIPN**). Extended π-conjugation in **4BGIPN** material results in yellow-green luminescence at 512 nm with a fast radiative rate of 5.5 × 10^−5^ s^−1^ and a photoluminescence quantum yield of 46% in methylcyclohexane solution. Such a nitrogen-rich **4BGIPN** material has a significantly stabilized highest occupied molecular orbital (HOMO) at −6.4 eV while the lowest unoccupied molecular orbital (LUMO) at −4.0 eV, indicating potential suitability for application as the electron transport layer or TADF class III emitter in OLEDs.

## Introduction

**Figure 1:**
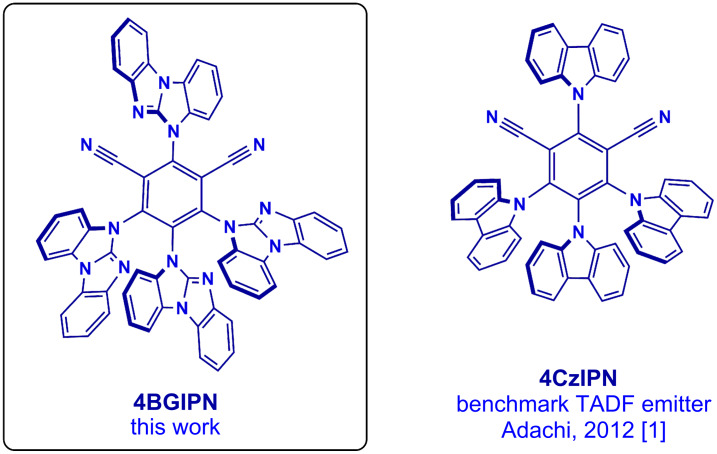
The molecular structures of the title compound **4BGIPN** and the benchmark TADF emitter **4CzIPN**.

Thermally activated delayed fluorescence (TADF) is a photoluminescence mechanism where excitons undergo thermally-assisted reverse-intersystem crossing from an excited triplet state to a higher-lying in energy singlet state to emit delayed fluorescence [1–3]. Organic TADF emitters have gained substantial attention in recent years for their prospective application in organic light-emitting diodes (OLEDs), photocatalysis, bioimaging, and sensors [4–6]. The ability to harvest both singlet and triplet excitons enable organic TADF emitters to compete with classic phosphorescent emitters that employ scarce metals such as iridium and platinum [7–9]. Since its first report in 2012 by Uoyama et al., 1,2,3,5-tetrakis(carbazol-9-yl)-4,6-dicyanobenzene (**4CzIPN**) has been a benchmark TADF emitter due to its high quantum yields and excellent performance in OLED devices [1]. **4CzIPN** is a donor–acceptor-type system where carbazole donor ligands are bound to the benzonitrile acceptor core moiety. In this work we have substituted the carbazole donors with 5*H*-benzo[*d*]benzo[4,5]imidazo[1,2-*a*]imidazole (benzoguanidine) ligands to give **4BGIPN**, see Figure 1. Benzoguanidine has an extended π-conjugation compared with carbazole and is more nitrogen-rich (three N-atoms vs one in carbazole). Thompson et al. recently reported a series of carbene–metal–amide (CMA) (metal = Cu, Ag, Au) emitters employing a benzoguanidine ligand [10]. The extended π-conjugation of benzoguanidine induced a larger hole–electron separation resulting in a smaller energy gap between the excited singlet and triplet states (S_1_ and T_1_) and Δ*E*_ST_ resulting in faster radiative rates. This study aimed to synthesize and explore the luminescent properties of the **4BGIPN** material containing a rigid benzoguanidine ligand in its molecular structure.

## Results and Discussion

### Synthesis and structure

**Figure 2:**
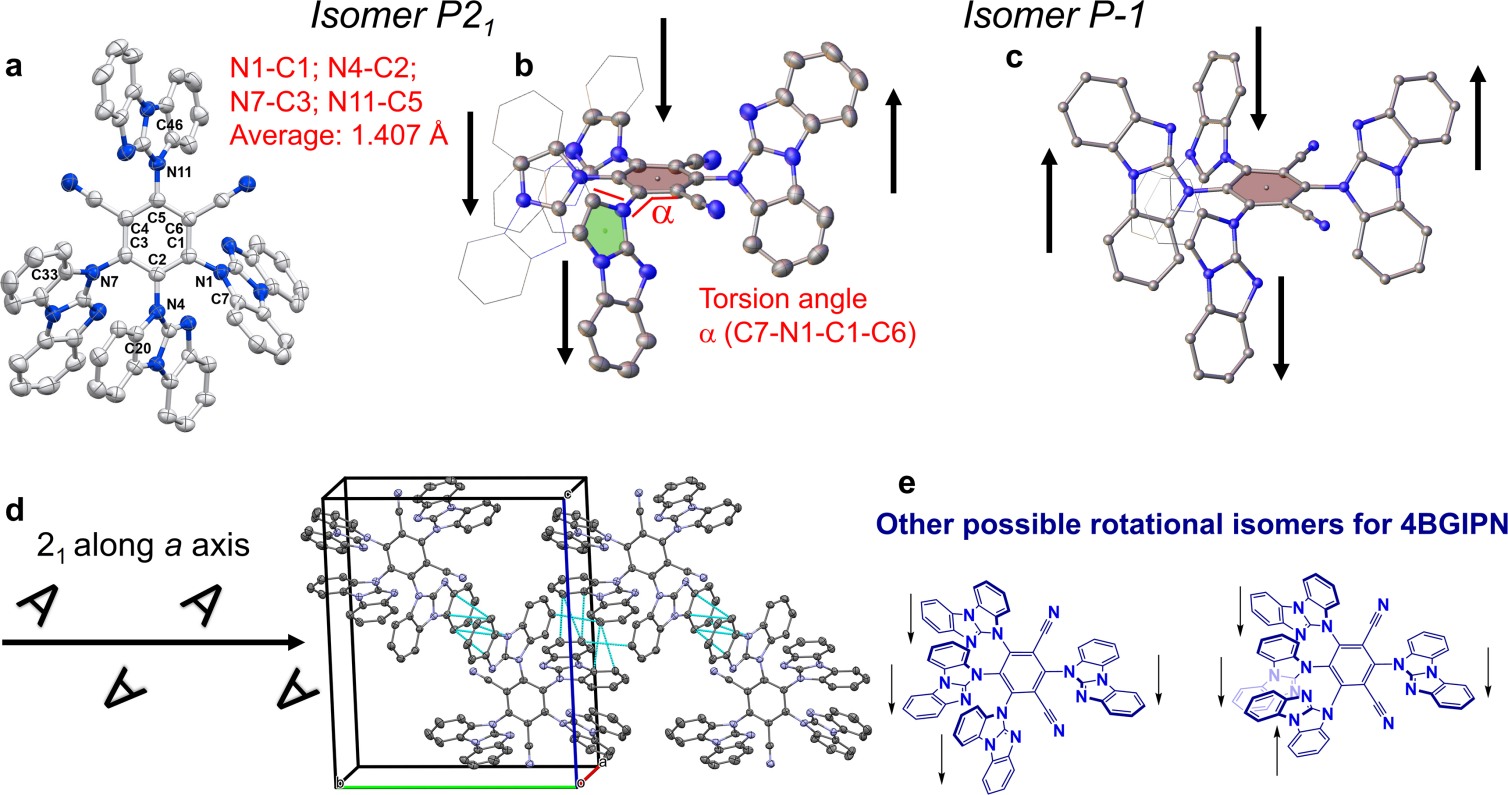
Crystal structure for compound **4BGIPN** in monoclinic form ((a) top view and (b) side view) where black arrows show opposite orientation of the benzoguanidine moieties around the central benzene ring. Representative example for the torsion angle α is shown in red; (c) Crystal structure for compound **4BGIPN** in triclinic form; (d) packing diagram with key geometrical parameters and intermolecular contacts shown as a cyan dashed line; (e) possible isomers for **4BGIPN** material. Ellipsoids are shown at the 50% level where hydrogen atoms are omitted for clarity.

**4BGIPN** was prepared in 70% yield by aromatic nucleophilic substitution reaction from 2,4,5,6-tetrafluoroisophthalonitrile and 5*H*-benzo[*d*]benzo[4,5]imidazo[1,2-*a*]imidazole (benzoguanidine) after deprotonation the latter with sodium hydride base. The compound shows poor solubility in most common organic solvents with moderate solubility in dichloromethane, 1,2-dichlorobenzene and dimethyl sulfoxide (DMSO). Compound **4BGIPN** was characterized by high-resolution mass spectrometry (HRMS), elemental analysis, and ^1^H/^13^C NMR spectroscopy. Proton NMR shows a complicated set of overlapping multiplets indicating that the reaction results in the formation of various isomers (rotamers) which are different by relative orientation of the benzoguanidine donor moieties with respect to each other (Figure 2, see Supporting Information File 1 for NMR). In DMSO-*d*_6_ solution, **4BGIPN** isomers do not show interconversion even upon warming to 120 °C, resulting in a similar set of signals. Excellent fit between HRMS and elemental analysis further supports the formation of the isomeric mixture of **4BGIPN** as evidenced by the identical molecular peak ion and C, H, N values within acceptable deviation of 0.4%. The decomposition temperature (*T*_d_, corresponds to 5% weight loss) was measured by thermogravimetric analysis (TGA) indicating excellent thermal stability for **4BGIPN** with *T*_d_ = 425 °C, which is similar to the benchmark material **4CzIPN**, having a *T*_d_ in the range of 402–449 °C (*T*_d_ range is dependent on the type of the **4CzIPN** polymorph) [11–12]. Differential scanning calorimetry (DSC) shows an endothermic process in the range of 237 to 265 °C which can be associated with the glass transition temperature (*T*_g_) for the isomeric mixture of **4BGIPN**. This is expectedly higher than the analogous *T*_g_ of 176 °C for the **4CzIPN** material [13] due to a lower molecular mass of the latter (Figure S1, Supporting Information File 1).

Single crystals for X-ray diffraction study were obtained by slow layer diffusion of hexanes into dichloromethane solution for **4BGIPN** at room temperature (Figure 2). The title compound crystallizes with two independent molecules in the unit cell of the triclinic (*P*−1, Figure 2c, yellow plates) and monoclinic chiral space group *P*2_1_ (Figure 2a,b,d, yellow blocks). Due to very weak reflection data, the structure of **4BGIPN** in triclinic form was refined in isotropic model, therefore, we do not discuss in detail the structural parameters. We only note that both forms are not due to the polymorphism but rather due to rotational isomerism of the **4BGIPN** material, i.e., different orientation of the benzoguanidine donor ligands above or below the central 4,6-dicyanobenzene ring. In the monoclinic form the two independent molecules of **4BGIPN** are related by a pseudo glide plane that do not completely align when superimposed through a glide operation. There is no evidence for systematic absences relating to the presence of a glide plane in the data supporting the refinement in the chiral *P*2_1_ space group. The structure was refined as a two-component inversion twin; the crystal structure as a whole is a racemic mixture of both orientations. The C_benzene_–N_benzoguanidine_ bond length varies within the error of the experiment from 1.402(5) to 1.420(5), giving an average of 1.407(13) Å for **4BGIPN**, which is closely similar to 1.405(8) Å reported for the benchmark **4CZIPN** compound.

Unlike carbazole, the benzoguanidine ligand lacks *C*_2_ rotational symmetry, thus enabling the benzoguanidine ligands to project above and below the plane of the central benzene ring. In both molecules in the asymmetric unit, the benzoguanidine moiety bound to the benzene carbon neighboring two nitrile groups, is orientated in the opposing projection about the plane of the benzene ring to the remaining benzoguanidine moieties (Figure 2b). Unlike monoclinic, the triclinic form of **4BGIPN** possesses two donor moieties pointing down at C1 and C3 carbon atoms while donor moieties at C2 and C5 are pointed up (Figure 2c). Several possible **4BGIPN** rotational isomers are demonstrated in Figure 2e, however, not isolated in this work. Compound **4BGIPN** possesses a twisted orientation between the donor (benzoguanidine) and acceptor (benzonitrile) ligands (Figure 2) due to steric hindrance imposed by benzoguanidine ligands and reflected by the torsion angle (α) laying in the range of 42.5(2)–64.3(2)º. We compared it with the more narrow torsion angle range of 55.1(2)–60.2(2)º for **4CzIPN** thus indicating that various carbazole donor ligands possess a very similar twist orientation [12]. The donor–acceptor twist angle has been demonstrated to be one of the key structural parameters enabling fast radiative rates for purely organic TADF materials since it’s directly related with the overlap integral between HOMO and LUMO orbitals and influences the energy differences between first singlet and triplet excited states [14]. Therefore, we expect a marked difference in the photophysical properties for **4BGIPN***,* vide infra.

Analysis of the intermolecular interactions revealed that **4BGIPN** molecules experience face to face intermolecular π–π stacking interactions between the benzoguanidine moieties similar to **4CzIPN** (reported by Etherington et al., [12]). The average interplanar distance for close neighbor benzoguanidine moieties in **4BGIPN** is 3.322(3) Å, which is significantly shorter (0.4 Å) than the 3.74(3) Å average distance between nearest neighbor carbazole ligands in **4CzIPN**, indicative for much stronger intermolecular interactions.

**Figure 3:**
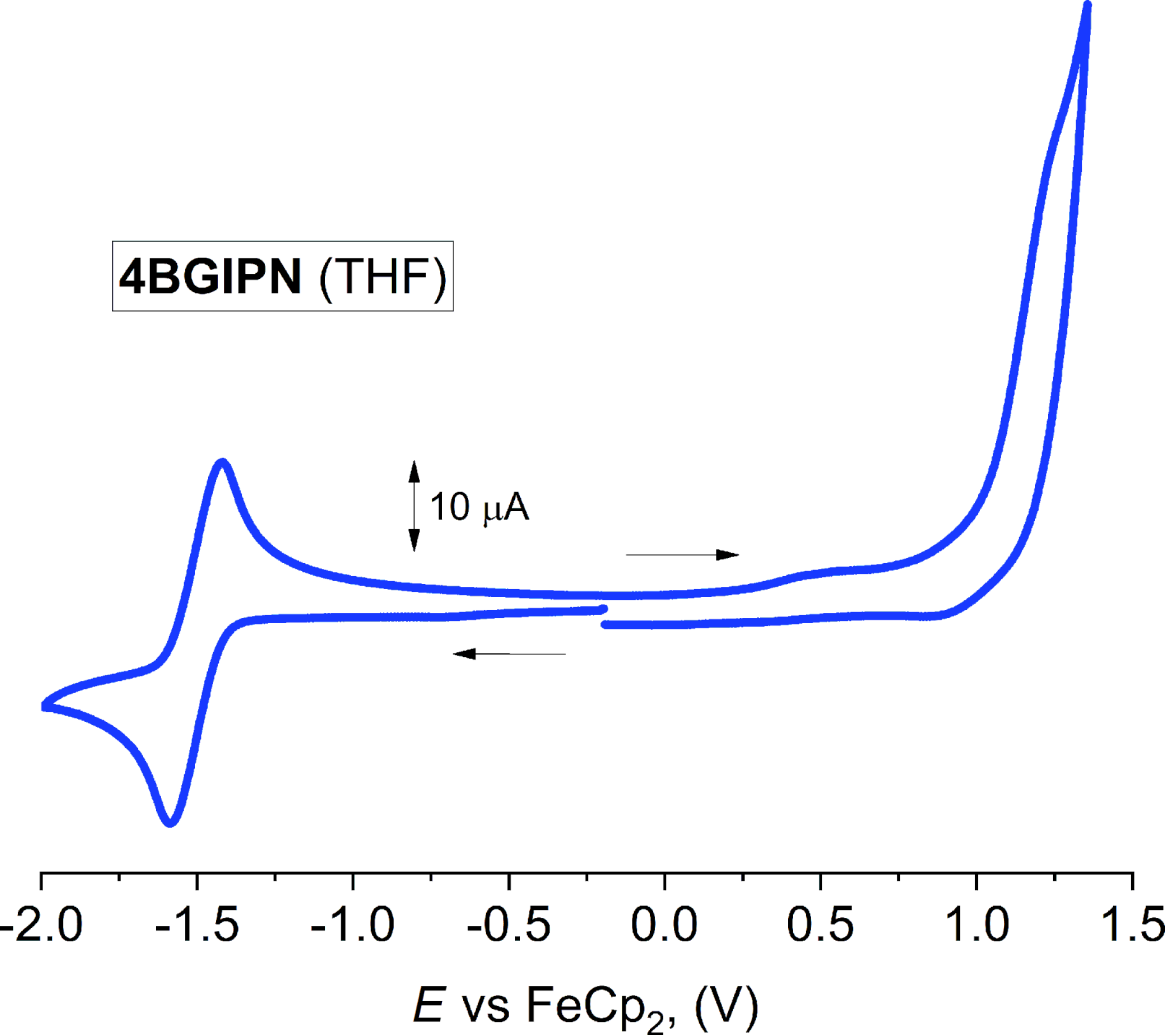
Full range cyclic voltammogram for **4BGIPN**. Recorded using a glassy carbon electrode in THF solution (1.4 mM) with [*n*-Bu_4_N]PF_6_ as supporting electrolyte (0.13 M), scan rate 0.1 Vs^−1^.

Cyclic voltammetry was used to analyze the redox behavior of **4BGIPN** in THF solution containing [*n*-Bu_4_N]PF_6_ as supporting electrolyte (Figure 3, Table 1). The reduction wave has a quasi-reversible character with the *E*_1/2_ at −1.50 V, which is 260 mV shifted to higher potential when compared with **4CzIPN** (−1.76 V) under similar conditions in THF [15]. Compounds **4BGIPN** and **4CzIPN** experience a reduction process at the benzonitrile core (see, the LUMO isosurface in Figure 6, vide infra). Therefore, the higher reduction potential for **4BGIPN** suggests that the benzonitrile core has a lower electron density, which is likely associated with extended π-conjugation and two additional electron withdrawing aza-type nitrogen atoms in the benzoguanidine moieties. This explains the ca. 0.2 eV more stabilized LUMO energy level for compound **4BGIPN** compared with **4CzIPN**. Both **4CzIPN** and **4BGIPN** exhibit an irreversible oxidation wave observed at +1.25 V for **4BGIPN** in THF and +0.94 V for **4CzIPN** in MeCN [15]. A higher oxidation potential (*E*_p_) for **4BGIPN** compared with the **4CzIPN** corroborates with the electron deficient nature of the benzoguanidine moiety thus making it harder to oxidize compared with the more electron-rich carbazole moiety. This results in stabilization of the HOMO energy level at −6.4 eV for **4BGIPN**. Significant stabilization for both HOMO and LUMO energy levels indicates the potential suitability of **4BGIPN** material for application not only as emitter in the emitting layer but also as an electron transport layer in the fabrication of OLEDs.

**Table 1:** Formal electrode potentials (peak position *E*_p_ for irreversible and *E*_1/2_ for quasi-reversible processes (*), *V*, vs FeCp_2_), onset potentials (*E, V*, vs FeCp_2_), peak-to-peak separation in parentheses for quasi-reversible processes (*ΔE*_p_ in mV), *E*_HOMO_/*E*_LUMO_ (eV) and band gap values (Δ*E*, eV) for the redox changes exhibited by **4BGIPN**.^a^

Complex	Reduction		*E*_LUMO_ eV^b^	Oxidation		*E*_HOMO_ eV^b^	Δ*E* eV
	
	*E*_1st_	*E*_onset red_		*E*_1st_	*E*_onset ox_		

**4BGIPN**	−1.50 (167)	−1.41	−3.98	+1.25	+1.01	−6.40	2.42

^a^In THF solution, recorded using a glassy carbon electrode, concentration 1.4 mM, supporting electrolyte [*n*-Bu_4_N][PF_6_] (0.13 M), measured at 0.1 V s^−1^. ^b^Calculated according to *E*_HOMO_* =* –(*E*_onset ox Fc/Fc_*_+ _+* 5.39) and *E*_LUMO_* =* –(*E*_onset red Fc/Fc+_* +* 5.39) eV.

### Photophysical properties and theoretical considerations

**Figure 4:**
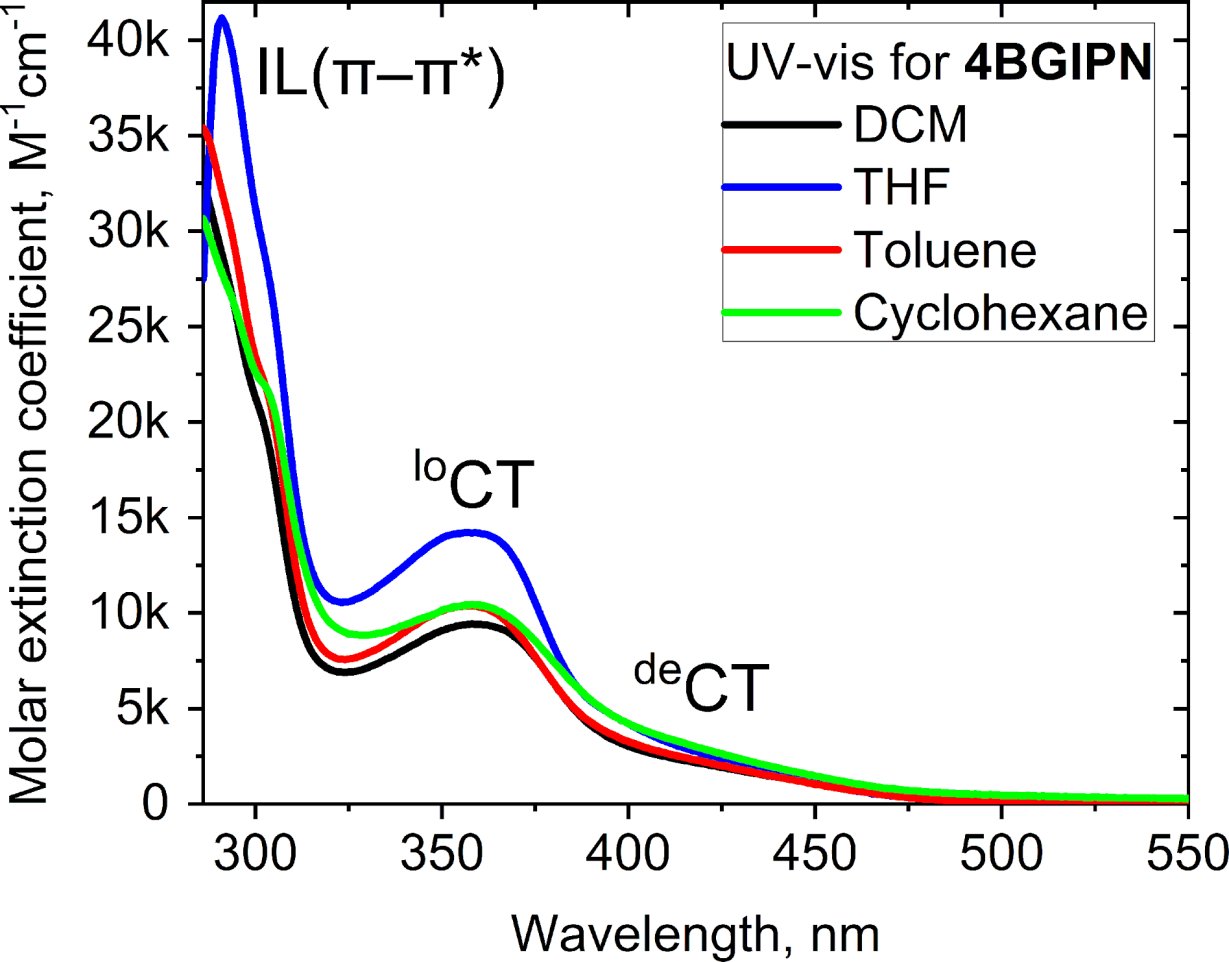
UV–vis absorption spectra for compound **4BGIPN** in various solvents.

**Figure 5:**
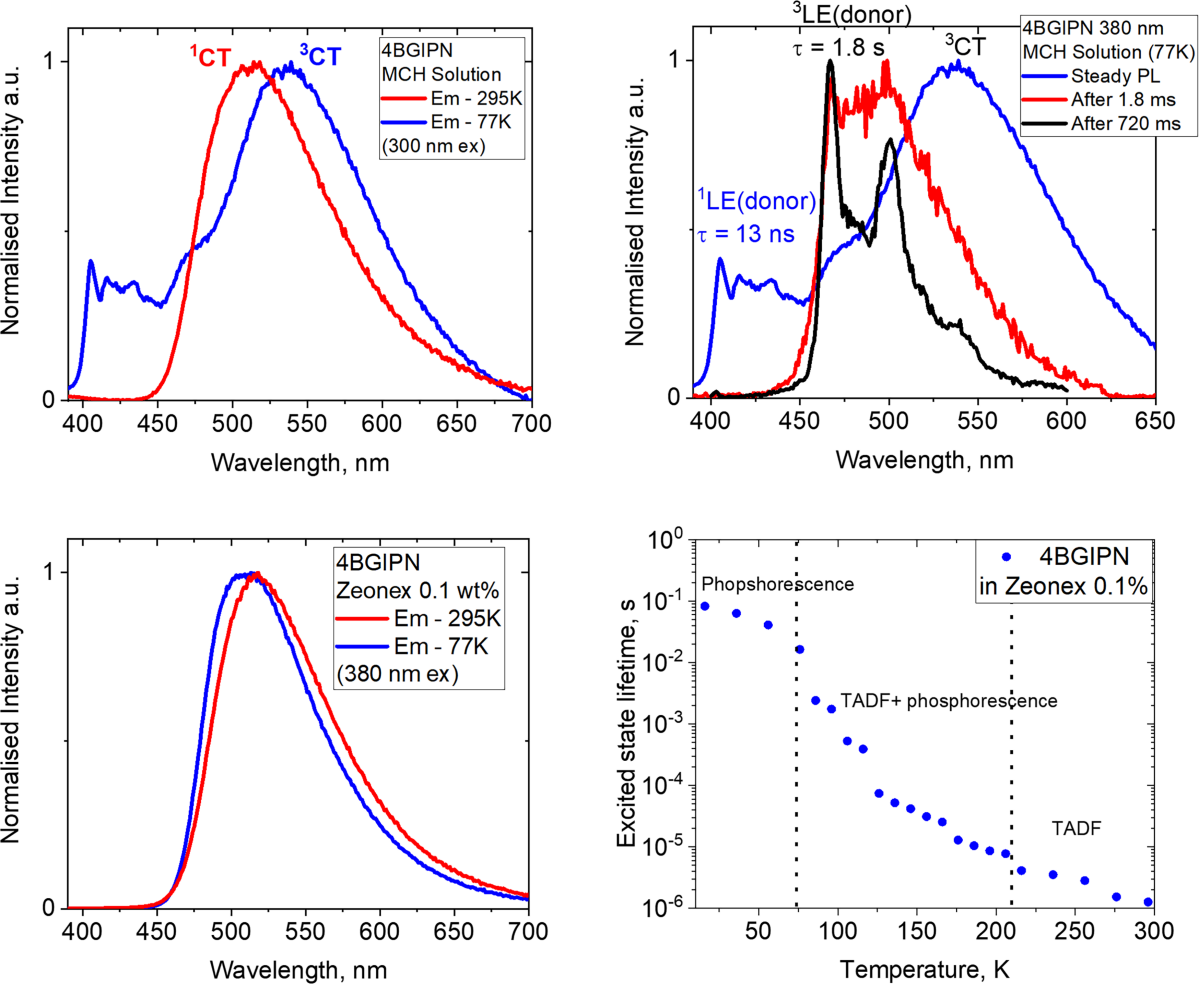
Photoluminescence spectra for **4BGIPN** at 295 and 77 K in (top left) MCH solution; (bottom left) Zeonex 0.1 wt % films; (top right) steady state and delayed PL in frozen glass MCH at 77 K with delays of 1.8 and 720 ms; (bottom right) excited state lifetime at various temperatures in the range from 16 to 296 K.

UV–vis and photoluminescence (PL) spectra for **4BGIPN** are shown in Figure 4 and Figure 5 while data in various media is collected in Table 2 and Table 3, respectively. UV–vis absorption spectra of **4BGIPN** show a strong π–π* intraligand transition (IL, benzoguanidine) at 290 nm with ε = 42000 M^−1^cm^−1^. Unlike **4CzIPN**, we do not observe any vibronically resolved carbazole absorption peaks which are commonly present at 325 nm [15]. Similar to **4CzIPN** [14–15], the UV–vis profile has two broad regions: localized charge transfer (^lo^CT) over 320–380 nm region with ε up to 14000 M^−1^ cm^−1^ and a delocalized charge transfer (^de^CT) broad shoulder over the region of ca. 380–460 nm with ε up to 1900 M^−1^ cm^−1^ (Table 2). Both ^lo^CT and ^de^CT bands are observed for the benchmark material **4CzIPN** [16] while originating from HOMO to LUMO transition in line with the theoretical calculations (Tables S1, S3, and S4, Supporting Information File 1). All CT bands experience a very weak solvatochromic effect with increasing solvent polarity from cyclohexane to dichloromethane. This indicates only minor change of the dipole moment upon vertical excitation from S_0_ (6.4 D) to S_1_ (7.2 D) excited states according to the TD-DFT theoretical calculations (Table S2, Supporting Information File 1).

**Table 2:** UV–vis data for compounds **4BGIPN** and **4CzIPN** [15,17] in various solvents.

	λ_abs_ [nm], (10^3^ ε/M^−1^ cm^−1^)			
	DCM	THF	toluene	cyclohexane

**4BGIPN**	436 sh (1.5) 358 (9.5)	435 sh (1.8) 357 (14.2)	439 sh (1.4) 358 (10.4)	439 sh (1.9) 358 (10.5)
**4CzIPN**	448 (7) [15] 378 [17]	438 (8) [15] –	441 (6) [15] 375 [17]	N/A

The photoluminescence (PL) characteristics of **4BGIPN** have been studied in methylcyclohexane solution (MCH, concentration 3.2 × 10^−5^ M) and Zeonex polymer films (0.1% concentration by weight) at 298 K and 77 K, which is shown in Figure 5 with data collected in Table 3. Compound **4BGIPN** exhibits a featureless yellow CT-type luminescence with λ_max_ = 525 nm that is 44 and 25 nm red-shifted compared to **4CzIPN** (λ_max_ = 481 and 500 nm in Zeonex and MCH, respectively) [12]. The solution photoluminescent quantum yield (PLQY) of **4BGIPN** is 46% under inert atmosphere and decreases down to 18% in aerated MCH solution. The reduction in quantum yield on exposure to oxygen is due to quenching of the triplet excited states indicating a TADF luminescence mechanism. PLQY in Zeonex films is 39% in air, which is lower than the PLQY of 87% reported for **4CzIPN** [17].

**Table 3:** Photophysical properties of **4BGIPN** in various media at 296 and 77 K.

	λ_em_ (nm)	τ (ns)	Φ (%)^a^	*k*_r_ (10^5^ s^−1^)^b^	*k*_nr_ (10^5^ s^−1^)^c^	^1^LE/^3^LE/^1^CT (eV)*^d^*	λ_em_ (nm)	τ

methylcyclohexane (MCH) solution 296 K						77 K		

**4BGIPN**	512	13 (50%) 1666 (50%)	46 (N_2_) (18 air)	5.48	6.43	3.12/2.73/2.70	405 (^1^LE);	13 ns
							470 (^3^LE);	1.8 s
							530 (^3^CT)	126 (36%) µs 1038 (64%) µs

0.1 wt % Zeonex matrix 296 K 16 K								

**4BGIPN**	517	12 (35%) 2023 (75%)	39	2.56	4.0	–/–/2.66	498	4.35 (31%) ms 36.8 (37%) ms 212 (32%) ms

^a^Absolute quantum yields determined using an integrating sphere; ^b^radiative rate constant *k*_r_ = Φ/τ; ^c^nonradiative constant *k*_nr_ = (1 – Φ)/τ; ^d^CT/LE energies based on the onset values of the emission spectra blue edge at 77 K and 295 K.

The two-component excited state lifetime with prompt and delayed fluorescence is characteristic for the TADF-type luminescence [1]. The excited state lifetime of **4BGIPN** has a biexponential decay with a prompt fluorescence τ_p_ = 13 ns and a delayed fluorescence τ_d_ = 1655 ns components in MCH solution. Zeonex films of **4BGIPN** exhibit a similar prompt τ_p_ = 12 ns, but an almost two-fold longer delayed fluorescence τ_d_ = 2.4 μs when compared to MCH solution. The archetype material **4CzIPN** possesses a similar prompt fluorescence component of 8 ns, whereas a delayed component is nearly ten-times longer, 8.9 and 8.8 μs, in MCH and Zeonex films, respectively [12]. These measurements correlate well with lower PLQY values for **4BGIPN** compared with **4CzIPN**, thus indicating that the use of a larger benzoguanidine donor ligand may open more nonradiative processes. This is reflected in the larger distribution in the torsion angles for **4BGIPN** compared with **4CzIPN**, vide supra.

We collected steady state luminescence and PL after long time delays for **4BGIPN** at 77 K to further support the assignment of the TADF mechanism and attempt to characterize LE and CT triplet states. The emission profiles experienced minor narrowing upon cooling to 77 K, while the PL profile remained broad and featureless (Figure 5) in Zeonex matices. Notably, a frozen MCH glass exhibits a new vibronically resolved component at 405 and 470 nm, together with a broad CT profile. The first resolved high-energy PL component at 405 nm (Figure 5 top right) has a lifetime of 13 ns, which we assigned to singlet locally excited fluorescence (^1^LE) from the benzoguanidine donor ligand. The second high-energy PL component at 470 nm becomes visible only after a long-time delay (720 ms, see Figure 5 black profile), therefore, we ascribed it to a phosphorescence from a higher lying ^3^LE state localized on a donor benzoguanidine moiety. Unlike ^1^LE-fluorescence, ^3^LE-phosphorescence has a very long lifetime of 1.8 s. Excitation spectra of the broad and resolved bands for **4BGIPN** in MCH glass at 77 K (Figure S4, Supporting Information File 1) follow a mirror image rule when compared with emission spectra showing both broad and resolved components, thus supporting the assignments of the ^3^CT and ^3^LE(donor) excited states. The excited state lifetime of the broad CT component has a multiexponential decay with averaged lifetimes of 0.7 ms in MCH glass and up to 212 ms in Zeonex films, which we assigned as phosphorescence from a ^3^CT state. A more than 100-fold increase in radiative lifetime on cooling to 77 K is characteristic for the organic TADF emitters [1]. Upon cooling Zeonex matrices of **4BGIPN** from 296 K to 16 K (Figure 5), the excited state lifetime shows an order of magnitude increase down to 60 K. However, only marginal increase of lifetime measured in the range of 60 to 16 K indicating a phosphorescence PL nature below 60 K.

**Figure 6:**
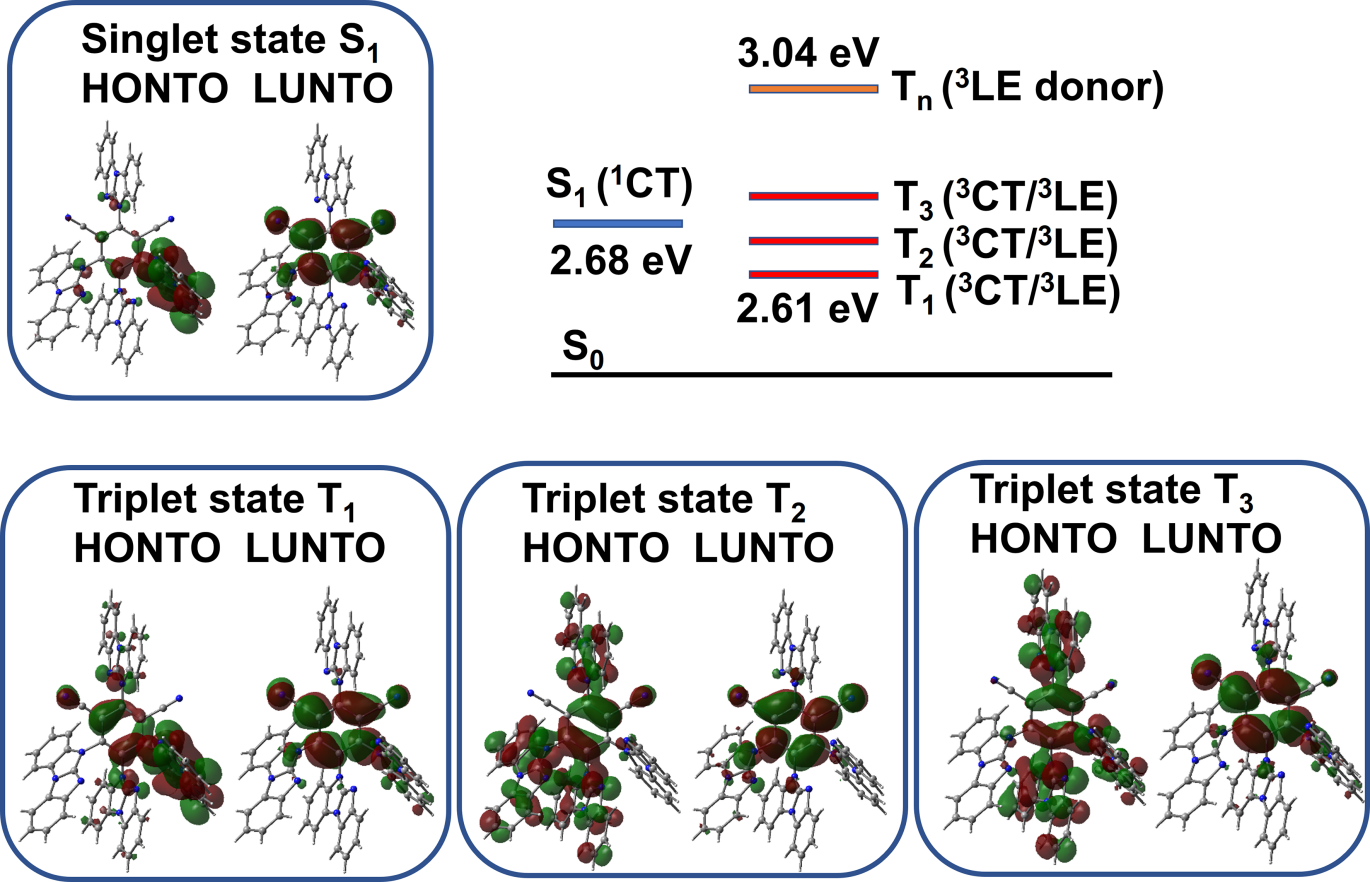
Energy state diagram and natural transition orbitals HONTO and LUNTO for compound **4BGIPN** in excited S_1_, T_1_, T_2_ and T_3_ states calculated from the crystal S_0_ geometry.

The charge transfer singlet (^1^CT) and local excited singlet (^1^LE), triplet (^3^LE) state energies were estimated from the onset values of the blue emission edge of the PL spectra at 295 K for ^1^CT and 720 ms delayed PL at 77 K for ^3^LE, respectively (Figure 5 and Table 3). It was expected to have a large energy difference between the states of similar character (LE) but different multiplicity, i.e., ^1^LE singlet state is 3.12 eV whereas ^3^LE state is 2.73 eV. The energy of the singlet ^1^CT state (2.70 eV) is only 0.03 eV lower compared to the energy of the ^3^LE state. Therefore, we ascribe compound **4BGIPN** to the class III TADF material where the ^3^LE is higher in energy than the manifold of the CT states as shown on Figure 6 [18]. The energy difference between singlet and triplet excited states is −0.03 eV for Δ*E*_1CT-3LE_. Such small energy Δ*E*_ST_ values further support the assignment of the TADF mechanism for the compound **4BGIPN**. Theoretical results (Tables S3 and S4, Supporting Information File 1) support our experimental observations, suggesting that low energy triplet states (T_1_, T_2_ and T_3_) possess a mixed CT/LE character with an energy difference up to 0.2 eV to the first singlet state S_1_. At the same time, class III TADF (*E*_3LE_ > *E*_CT_) [19] and class II TADF materials (*E*_3LE_ ≈ *E*_CT_) [20] are reported to have shorter excited state lifetimes compared with class I TADF materials, for instance **4CzIPN** (Δ*E*_1CT-3LE_ = +0.09 eV [12]). The short 1.6 microsecond excited state lifetime for **4BGIPN** (regardless of somewhat lower PLQY) is in line with those reported for other TADF class III systems [19]. We have been unable to resolve the ^3^LE state for **4BGIPN** in Zeonex matrices regardless of numerous efforts and cooling the films down to 16 K and applying long time delays.

## Conclusion

We have synthesized and characterized a donor–acceptor-type thermally activated delayed fluorescent emitter **4BGIPN** with four terminal benzoguanidine donor moieties surrounding the benzonitrile acceptor core. We found that the material is formed as a mixture of the rotational isomers that do not experience interconversion upon heating the **4BGIPN** solution in DMSO to 120 °C. Two rotational isomers were successfully crystallized to show different up and down orientations of the benzoguanidine donor ligands around the central 4,6-dicyanobenzene core. Unlike the **4CzIPN** compound, the **4BGIPN** emitter can crystallize in a chiral *P*2_1_ space group due to the parallel and antiparallel orientation of the benzoguanidine donors with respect to each other, lack of *C*_2_ rotational symmetry and extended π-conjugation. The twisted structure of the **4BGIPN** ensures that the donor groups accommodate the highest occupied molecular orbital (HOMO) while the acceptor 4,6-dicyanobenzene moiety contains the lowest unoccupied molecular orbital (LUMO) and is supported by the TD-DFT calculations. A comparison of the electronic parameters between benchmark **4CzIPN** and new **4BGIPN** materials revealed that benzoguanidine acts as a weaker donor ligand compared with carbazole, resulting in greater stabilization of the HOMO energy level down to −6.4 eV rather than LUMO. The significant stabilization of both HOMO and LUMO energy levels, along with multiple electron-withdrawing aza-nitrogen atoms in the structure of **4BGIPN**, suggests its potential suitability as an electron transport layer in OLED (organic light-emitting diode) devices. Variable temperature photoluminescence studies revealed that **4BGIPN** corresponds to the class III TADF system (*E*_3LE_ > *E*_1CT_), while having a small energy difference between singlet and triplet excited states of −0.03 eV for *ΔE*_1CT-3LE_. Theoretical calculations support that the first three triplet excited states possess a mixed CT/LE character while benzoguanidine singlet ^1^LE state is up to 0.4 eV higher in energy than the singlet ^1^CT state. The high quantum yields of up to 46% indicate that the yellow-green **4BGIPN** emitter shows a high promise as a platform for developing bright **4BGIPN**-TADF class III type compounds with unity PLQY. Future works may benefit in isolating a particular isomer that could show superior photophysical TADF characteristics important for fabricating TADF OLED devices with improved operating stability.

## Experimental

### General considerations

All reactions were performed under a N_2_ atmosphere. Solvents were dried as required. Sodium hydride was washed from mineral oil with diethyl ether and dried prior to use. 5*H*-Benzo[*d*]benzo[4,5]imidazo[1,2-*a*]imidazole (benzoguanidine) was obtained according to the literature protocol [10] while 2,4,5,6-tetrafluoroisophthalonitrile was purchased from Fluorochem Ltd. and used as received. ^1^H and ^13^C{^1^H} NMR spectra were recorded using a Bruker AVIII HD 500 MHz NMR spectrometer. ^1^H NMR spectra (500.19 MHz) and ^13^C{^1^H} (125.79 MHz) were referenced to dichloromethane-*d*_2_ at δ 5.32 (^13^C, δ 53.84). All electrochemical experiments were performed using an Autolab PGSTAT 302N computer-controlled potentiostat. Cyclic voltammetry (CV) was performed using a three-electrode configuration consisting of a glassy carbon macrodisk working electrode (GCE) (diameter of 3 mm; BASi, Indiana, U.S.A.) combined with a Pt wire counter electrode (99.99%; GoodFellow, Cambridge, U.K.) and an Ag wire pseudoreference electrode (99.99%; GoodFellow, Cambridge, U.K.). The GCE was polished between experiments using alumina slurry (0.3 μm), rinsed in distilled water and subjected to brief sonication to remove any adhering alumina microparticles. The metal electrodes were then dried in an oven at 100 °C to remove residual traces of water, the GCE was left to air dry and residual traces of water were removed under vacuum. The Ag wire pseudoreference electrodes were calibrated to the ferrocene/ferrocenium couple in THF at the end of each run to allow for any drift in potential, following IUPAC recommendations [16]. All electrochemical measurements were performed at ambient temperature under an inert N_2_ atmosphere in THF containing the complex under study (0.14 mM) and the supporting electrolyte [*n*-Bu_4_N][PF_6_] (0.13 mM). Data were recorded with Autolab NOVA software (v. 1.11). Thermogravimetric analysis was performed by the Microanalysis Laboratory at the University of Manchester. Mass spectrometry data were obtained by the Mass Spectrometry Laboratory at the University of Manchester.

**Synthesis of 4BGIPN.** 5*H*-Benzo[*d*]benzo[4,5]imidazo[1,2-*a*]imidazole (benzoguanidine, [10]) (700 mg, 3.38 mmol) was added to a suspension of NaH (81.0 mg, 3.38 mmol) in anhydrous DMF (40 mL) at 0 °C under a stream of N_2_. The reaction mixture was stirred for 1 h at room temperature. 2,4,5,6-Tetrafluoroisophthalonitrile (135 mg, 676 μmol) was added to the reaction mixture under N_2_. The reaction mixture was heated to 140 °C and left to stir overnight. The reaction mixture was dried under vacuum to remove DMF. The crude product was extracted with DCM and washed with water. The organic phase was collected and dried with MgSO_4_, filtered and concentrated under vacuo. The product was further purified by column chromatography (ethyl acetate/hexane 1:4) to give the pure product as a yellow powder in 70% yield (450 mg, 474 μmol). Single crystals suitable for X-ray diffraction were grown by layering a concentrated solution in DCM with hexane which was left for slow evaporation. ^1^H NMR (700 MHz, DMSO-*d*_6_) δ 8.46–8.35 (m), 8.23–8.22 (m), 8.16–8.06 (m), 7.94–6.86 (m), 6.82–6.80 (m), 6.59–6.54 (m), 6.47–6.41 (m), 6.35–6.29 (m), 6.24–6.23 (d, *J* = 7.9 Hz); ^13^C{^1^H} NMR (126 MHz, CD_2_Cl_2_) δ 151.17, 149.67, 146.84, 146.65, 146.27, 142.44, 142.29, 142.01, 134.45, 133.78, 133.00, 132.63, 131.91, 130.98, 128.91, 128.50, 127.82, 126.78, 126.58, 126.41, 126.24, 125.84, 124.85, 124.51, 124.39, 124.22, 124.11, 124.01, 123.82, 123.71, 123.53, 123.38, 123.30, 123.23, 122.97, 122.76, 121.99, 121.91, 121.61, 121.41, 121.28, 120.01, 119.91, 119.68, 119.43, 119.16, 117.13, 112.60, 112.41, 112.06, 112.00, 111.69, 111.33, 111.21, 111.11, 110.88, 110.82, 110.74, 110.44, 110.17 ppm; Anal. calcd. for C_60_H_32_N_14_ (948.29): C, 75.94; H, 3.40; N, 20.66; found: C, 75.59; H, 3.54; N, 20.28; HRESIMS *m*/*z*: [M + Na]^+^ calcd. for C_60_H_32_N_14_Na, 971.2827; found, 971.2854.

### X-ray crystallography

Crystals suitable for X-ray diffraction study were obtained by slow layer diffusion of hexanes/petroleum ether into dichloromethane solution for **4BGIPN** at room temperature. Crystals were mounted in oil on a MiTeGen loop and fixed on the diffractometer in a cold nitrogen stream. Data were collected using dual wavelength Rigaku FR-X rotating anode diffractometer using Cu Kα (λ = 1.54146 Å) radiation, equipped with an AFC-11 4-circle kappa goniometer, VariMAX^TM^ microfocus optics, a Hypix-6000HE detector and an Oxford Cryosystems 800 plus nitrogen flow gas system, at a temperature of 100 K. Data were collected and reduced using CrysAlisPro v42 [21–22]. Absorption correction was performed using empirical methods (SCALE3 ABSPACK) based upon symmetry-equivalent reflections combined with measurements at different azimuthal angles.

For the final refinement, the contribution of severely disordered CH_2_Cl_2_ molecules in the crystals of **4BGIPN** was accounted for by applying a solvent void mask calculated using BYPASS, implemented through Olex2 [23]. Structures were solved by direct method/intrinsic phasing and refined by the full-matrix least-squares against F^2^_._ All non-hydrogen atoms were refined with anisotropic atomic displacement parameters. All hydrogen atoms were positioned geometrically and constrained to ride on their parent atoms with C–H = 0.95–1.00 Å, and U_iso_ = 1.2–1.5 U_eq_ (parent atom). All calculations were performed using the SHELXL software and Olex2 graphical user interface [22–23].

**4BGIPN (monoclinic *P*2_1_)**, CCDC number 2243340, C_60_H_32_N_14_, monoclinic, space group *P*2_1_ (no. 4), *a* = 17.4217(4) Å, *b* = 15.2552(3) Å, *c* = 20.8314(6) Å, β = 114.583(3)°, *V* = 5034.6(2) Å^3^, *Z* = 4, *d*_calc_ = 1.252 g cm**^−^**^3^, μ = 0.623 mm**^−^**^1^, yellow block, 33714 reflections measured (4.664° ≤ 2Θ ≤ 152.79°), 17837 unique (*R*_int_ = 0.0314, *R*_sigma_ = 0.0512) which were used in all calculations. The final *R*_1_ was 0.0462 (I > 2σ(I)) and *wR*_2_ was 0.1190 (all data). *GOF* = 1.042, Δρ_min_/Δρ_max_ = 0.4/−0.2 e Å^−3^.

**4BGIPN (triclinic *P*−1)**, CCDC number 2287367, C_60_H_32_N_14_·0.5CH_2_Cl_2_ (*M* = 991.46 g/mol): triclinic, space group *P*−1 (no. 2), *a* = 12.8390(16) Å, *b* = 21.536(3) Å, *c* = 23.149(5) Å, α = 65.321(17)°, β = 82.509(14)°, γ = 89.955(11)°, *V* = 5756.1(19) Å^3^, *Z* = 4, *T* = 100.00(13) K, μ(Cu Kα) = 0.981 mm^−1^, *d*_calc_ = 1.144 g/cm^3^, 68407 reflections measured (4.244° ≤ 2Θ ≤ 151.924°), 22637 unique (*R*_int_ = 0.1849, *R*_sigma_ = 0.1991) which were used in all calculations. The final *R*_1_ was 0.2870 (I > 2σ(I)) and *wR*_2_ was 0.6519 (all data).

### Computational results

Computations were performed using density functional theory (DFT) for the ground state and time-dependent DFT (TD-DFT) with Tamm–Dancoff approximation [24–25] for the excited states calculations, using the global hybrid MN15 functional by Truhlar [26] in combination with the def2-TZVP basis set by Ahlrichs [27–28]. TD-DFT calculations were performed to elucidate the nature of the excited state in a crystalline and optimized molecular geometry of **4BGIPN** with all data collected in Supporting Information File 1 (Tables S1–S4). All calculations were carried out by Gaussian 16 [29] and HOMO–LUMO overlap integrals were calculated using Multiwfn program [30].

## Supporting Information

File 1 Additional experimental data.

File 2 CIF file.
