# Supplementary material for: Organic thermally activated delayed fluorescence material with strained benzoguanidine donor
Source: Beilstein J Org Chem. 2023 Sep 7;19:1289–98. doi: 10.3762/bjoc.19.95 (PMC10494236; doi:10.3762/bjoc.19.95)
Supplement: File 1 — Additional experimental data. [file Beilstein_J_Org_Chem-19-1289-s001.pdf]

BEILSTEIN JOURNAL OF ORGANIC CHEMISTRY

Supporting Information

for

Organic thermally activated delayed fluorescence material with strained benzoguanidine donor

Alexander C. Brannan, Elvie F. P. Beaumont, Nguyen Le Phuoc,
George F. S. Whitehead, Mikko Linnolahti and Alexander S. Romanov

Beilstein J. Org. Chem. **2023,** *19,* 1289–1298. doi:10.3762/bjoc.19.95

Additional experimental data

License and Terms: This is a supporting information file under the terms of the Creative Commons Attribution License (https://creativecommons.org/licenses/by/4.0). Please note that the reuse, redistribution and reproduction in particular requires that the author(s) and source are credited and that individual graphics may be subject to special legal provisions.
The license is subject to the *Beilstein Journal of Organic Chemistry* terms and conditions: (https://www.beilstein-journals.org/bjoc/terms)

^{1}H and ^{13}C NMR, HRMS spectra for **4BGIPN** **S**2–S4

Figure S1. Thermogravimetric analysis (top) and differential scanning colourimetry for **4BGIPN** S5

Figure S2. An overlay of the X-ray crystal structure and optimised S_0 geometry of compound **4BGIPN** **S**6

Figure S3. The deconvolution and estimation of the experimental value for the oscillator strength coefficients associated with the **4BGIPN**. S6

Figure S4. Excitation and emission spectra for **4BGIPN** in frozen MCH glass at 77 K S7

Photophysical characterisation S8

Table S1. HOMO and LUMO molecular orbitals S8

Table S2. Dipole moments in S0 and S1 states in S0 geometry S9

Table S3. Vertical excitations $S_0 \rightarrow S_1$ and $S_0 \rightarrow T_n$, character and oscillator strength coefficients for $S_0 \rightarrow S_1$. S9

Table S4. HONTO andd LUNTO molecular orbitals of the excited states. S10

Coordinates for compound **4BGIPN** S12

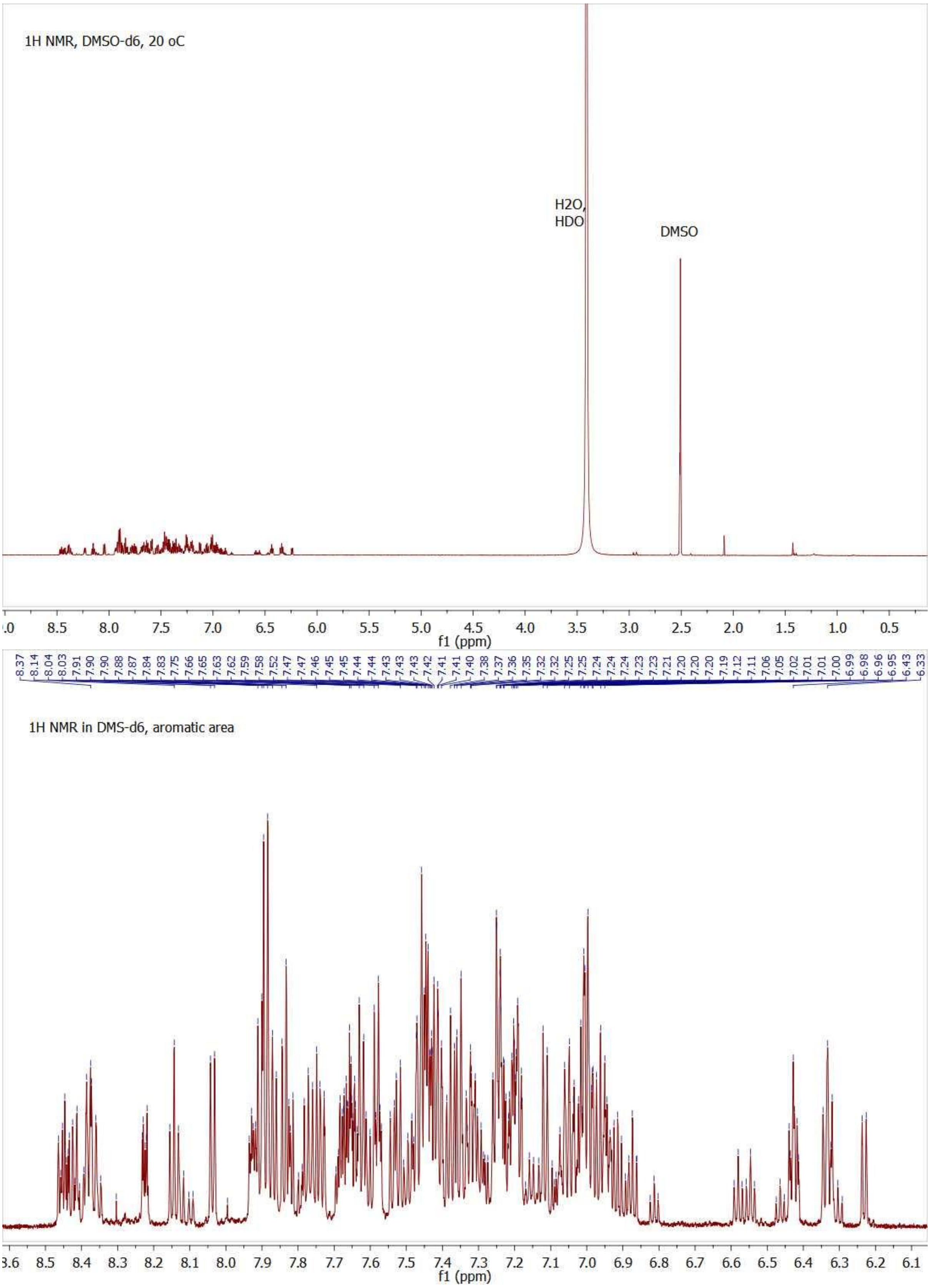

^{1}H NMR (700 MHz, DMSO-d_6) spectrum of **4BGIPN**.

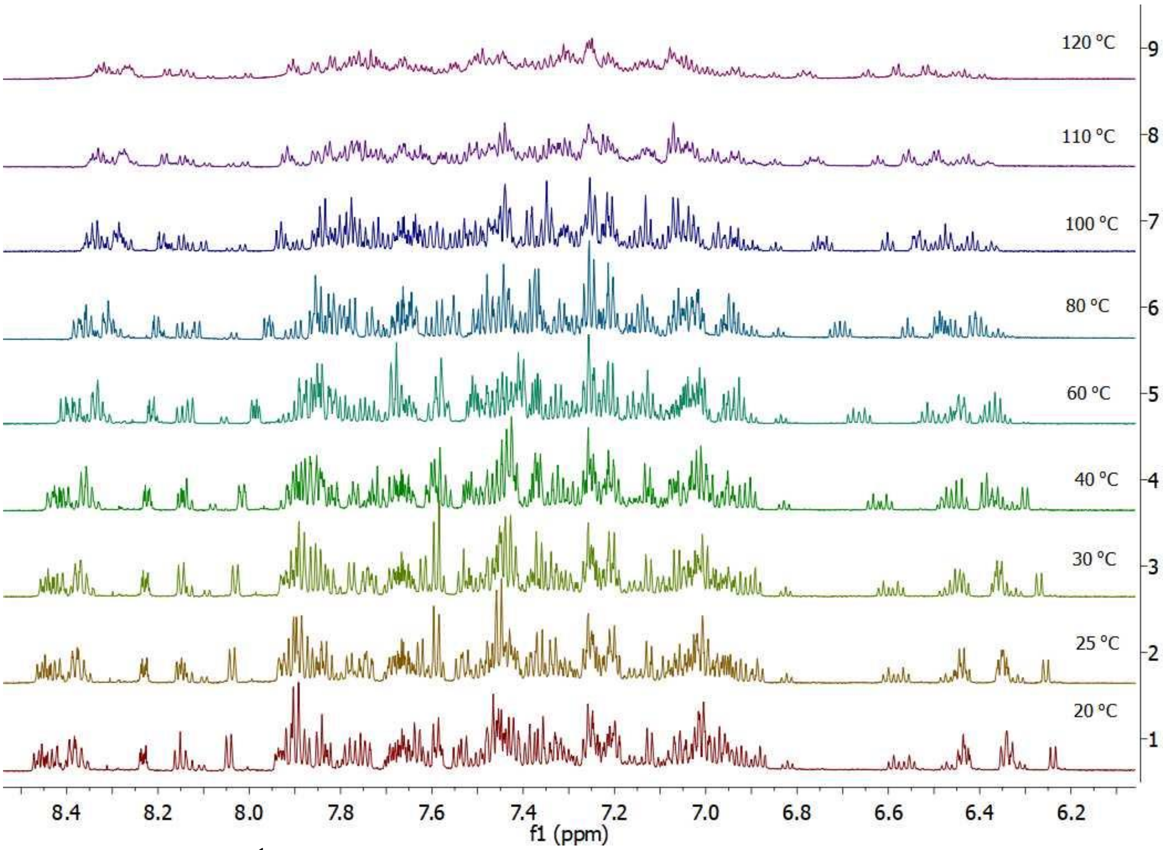

Varied temperature ^{1}H NMR (700 MHz, DMSO-d_6) spectra of **4BGIPN** between 20 and 120 ^{o}C.

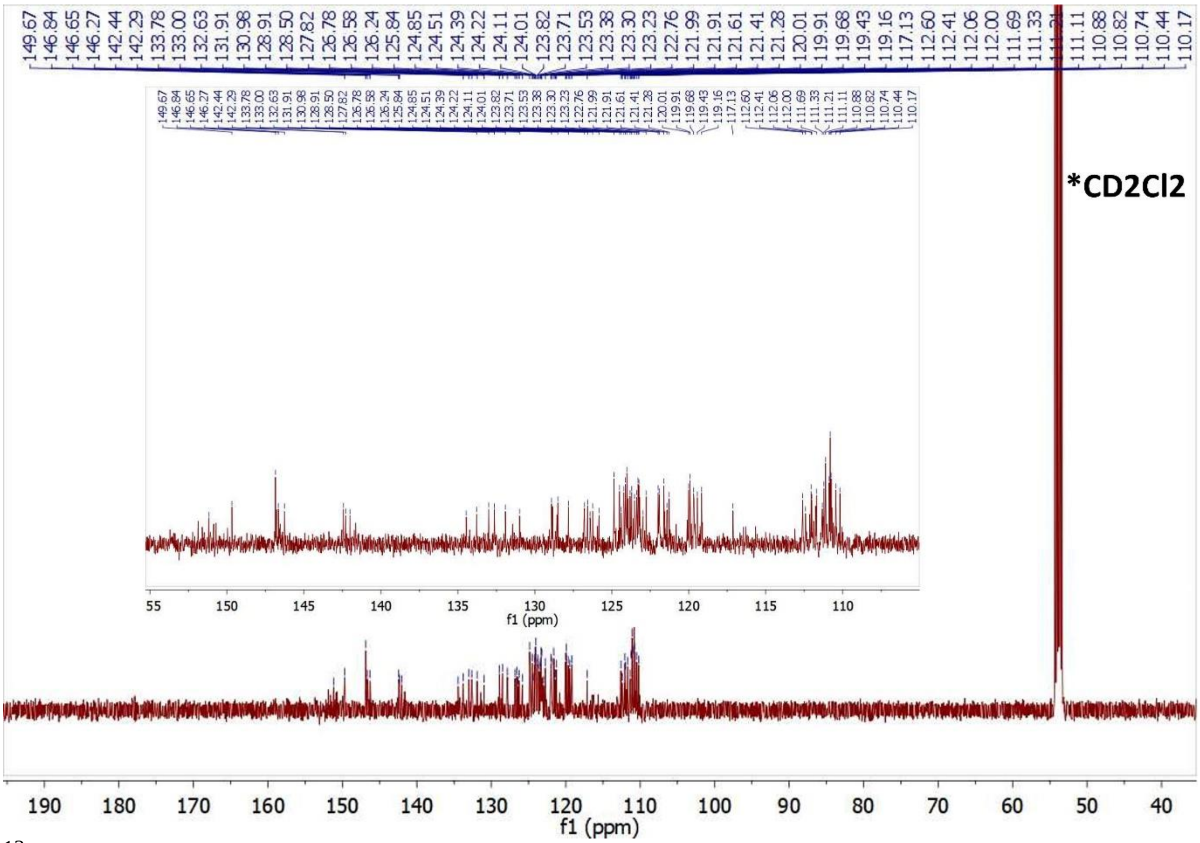

^{13}C NMR (DCM-d_2) spectrum of **4BGIPN**

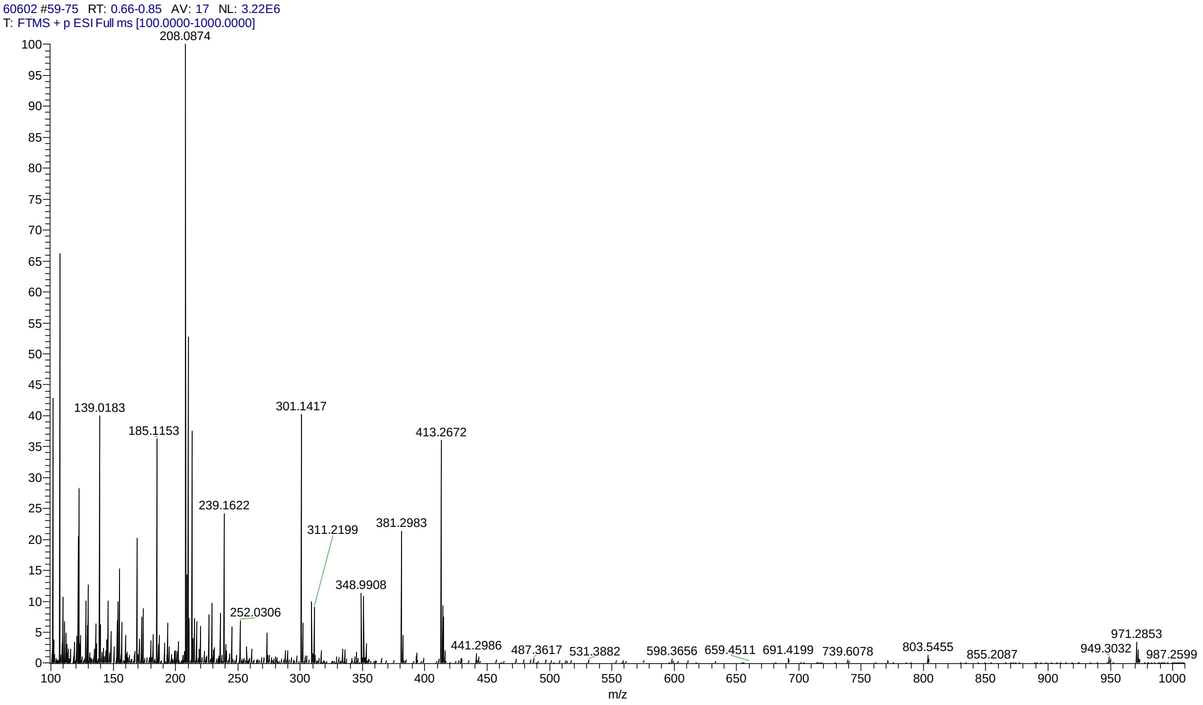

Full High-Resolution Mass Spectrometry (HRMS).

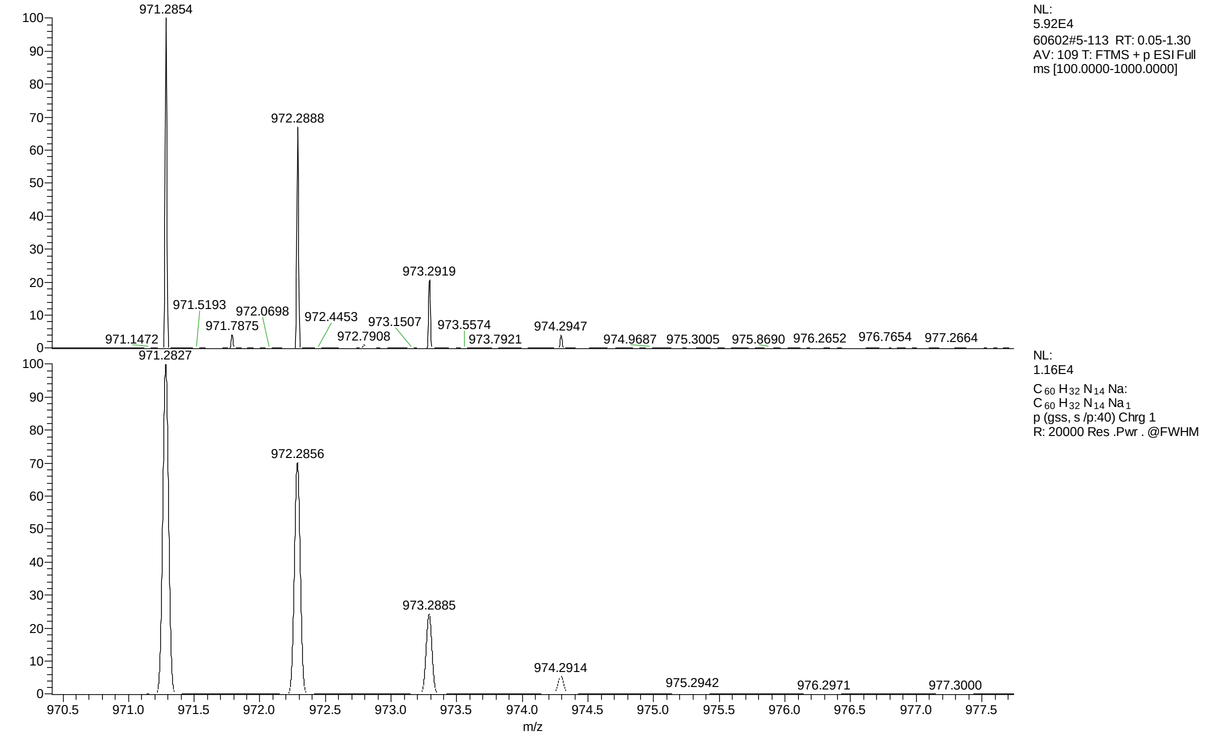

HRMS Thermo Executive Plus EMR Orbitrab HESI POS and NEG.

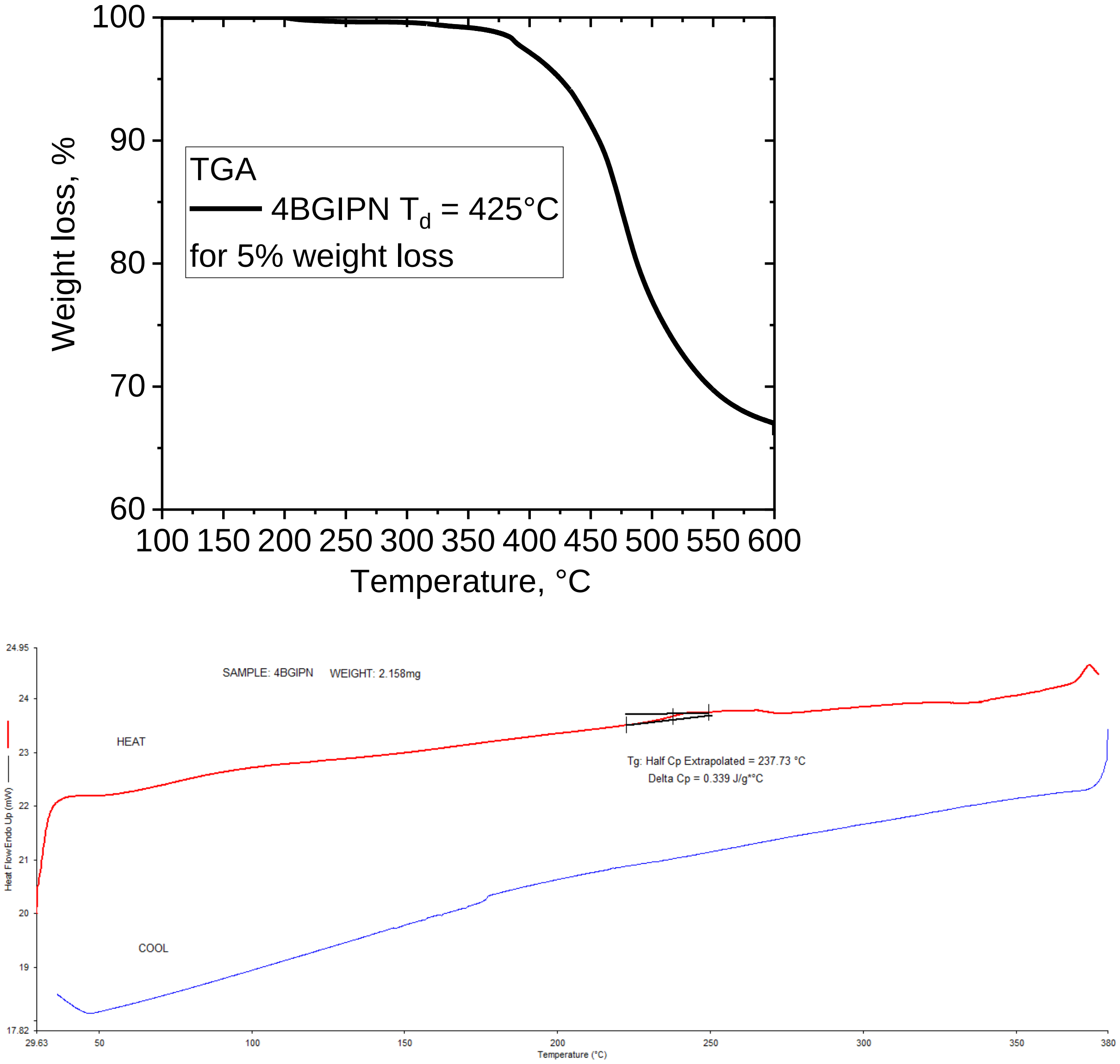

Figure S1. Thermogravimetric analysis (top) and differential scanning colourimetry (bottom, glass transition temperature is in the range 237–260 ºC) for isomer mixture of compound **4BGIPN**.

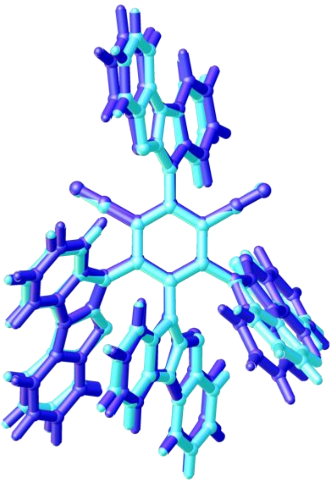

Figure S2. An overlay of the X-ray crystal structure and optimised S_0 geometry of compound **4BGIPN**.

Model	Gaussian								
Equation	y = y0 + A/(w*sqrt(pi/(4*ln(2)))) * exp(-4*ln(2)*(x-xc)^2/w^2)								
Plot	Peak1(MCH)	Peak2(MCH)	Peak3(MCH)	Peak4(MCH)	Peak5(MCH)	Peak6(MCH)	Peak7(MCH)	Peak8(MCH)	Peak9(MCH)
y0	323.55782 ± 542.54455	323.55782 ± 542.54455	323.55782 ± 542.54455	323.55782 ± 542.54455	323.55782 ± 542.54455	323.55782 ± 542.54455	323.55782 ± 542.54455	323.55782 ± 542.54455	323.55782 ± 542.54455
xc	45009.56938 ± 74.1632	41837.32057 ± 341.6130	39287.08134 ± 67785.99	38172.2488 ± 42020.9002	37009.56938 ± 119108.71	35263.15789 ± 9552.907	32851.67464 ± 701.1128	28023.92344 ± 267.3550	25028.70813 ± 650.4402
A	1.28225E8 ± 1.06965E	1.47944E8 ± 6.29381E7	1.03193E8 ± 1.54135E10	9.45854E7 ± 3.52137E10	8.26841E7 ± 2.1158E10	6.46344E7 ± 9.86407E8	4.33349E7 ± 3.51813E7	2.11571E7 ± 6.46008E6	8.02609E6 ± 5.97236E6
w	1943.46779 ± 191.0462	1943.46779 ± 531.23031	1943.46779 ± 32253.664	1943.46779 ± 181291.447	1943.46779 ± 89256.5861	1943.46779 ± 8445.8429	1943.46779 ± 989.48591	1943.46779 ± 686.0517	1943.46779 ± 1625.9481
Reduced Chi-Sqr	8.32793E7								
R-Square (COD)	0.74652								
Adj. R-Square	0.73206								

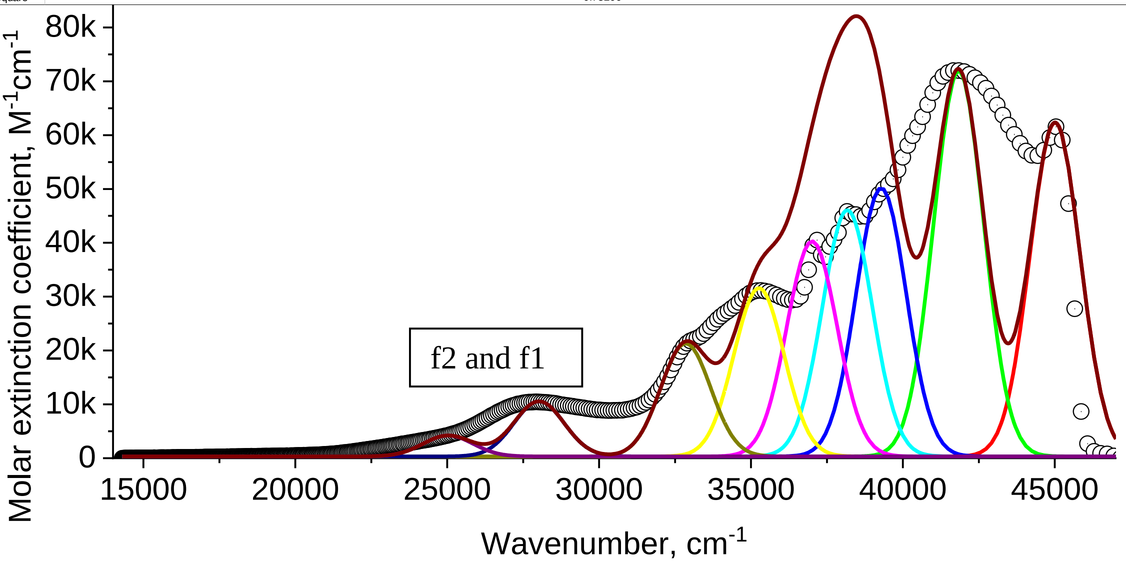

Figure S3. The deconvolution and estimation of the experimental value for the oscillator strength coefficients associated with the ^{lo}CT and ^{de}CT bands:

$f1 = 4{,}32 \cdot 10^{-9} \int \varepsilon(\bar{\nu})\, d(\bar{\nu}) = 4{,}32 \cdot 10^{-9} \cdot 2.1 \cdot 10^{7} (Peak8) = 0.091\ while\ f2 =$ 0.034 based on peak 9. The values are close similar with the theoretically calculated values *f*1 = 0.091 while *f*2 = 0.019.

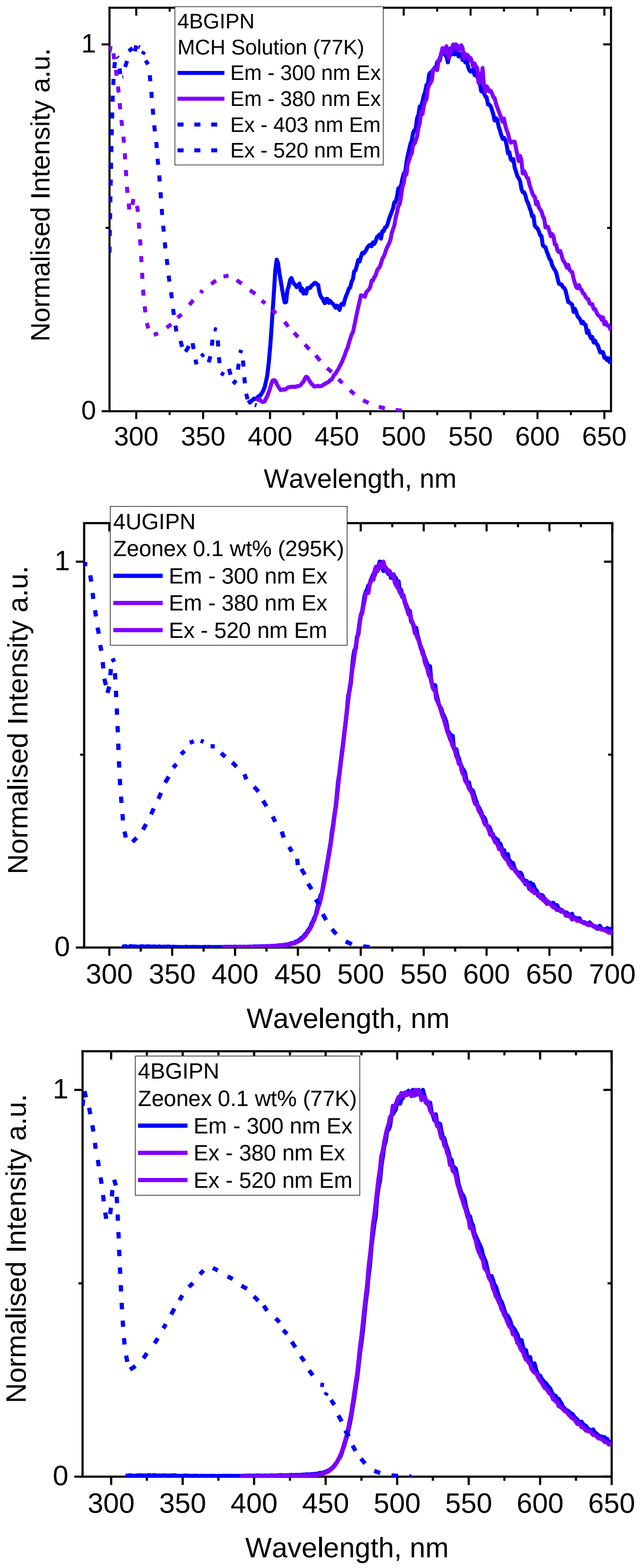

Figure S4. Excitation and emission spectra for **4BGIPN** in frozen MCH glass at 77 K (top); Zeonex 0.1 wt % films at 295 K (middle) and 77 K (bottom) with emission and excitation wavelengths in legend.

Photophysical characterisation.

UV-visible absorption spectra were recorded using a Varian Cary 5000 UV-Vis-NIR spectrometer. Photoluminescence measurements were recorded on an Edinburgh Instruments FLS-1000 spectrometer with a solids mount attachment where appropriate. Absolute photoluminescence quantum yields were recorded using Hamamatsu Quantaurus-QY C11347-11. Quantum yields have been measured in air for solid samples and under nitrogen for solutions. Time resolved luminescence data were collected on a time-correlated single photon counting (TCSPC) Edinburgh Instruments FLS-1000 spectrometer using F-900 software. A xenon flash lamp and EPL pulsed diode lasers were used as excitation sources. The collected data were analysed using F-900 software.

Computational details.

Table S1. HOMO and LUMO molecular orbitals in crystal and optimized S_0 geometry.

	HOMO	LUMO
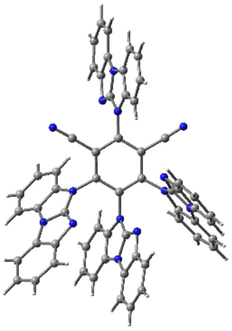 (crystal)<br>Overlap integral: 0.27	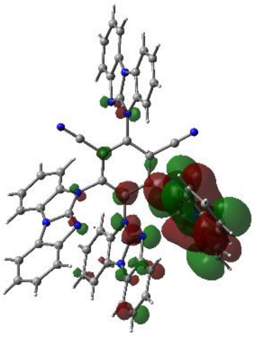	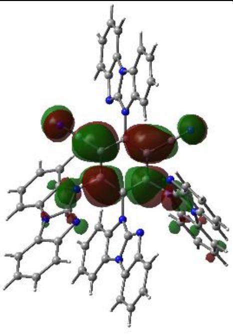
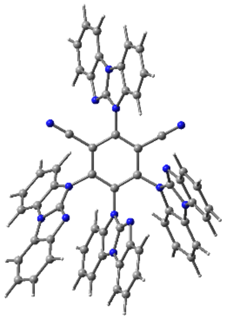 (opt)<br>Overlap integral: 0.29	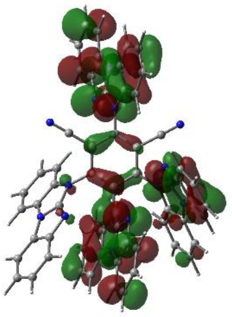	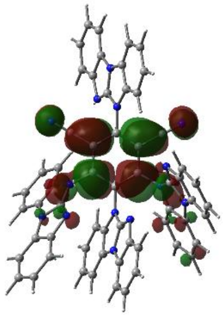

Tables S2. Dipole moments in S_0 and S_1 states in crystal and optimized S_0 geometry.

	S_0	S_1
(crystal)	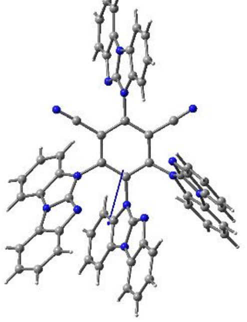<br>6.7D	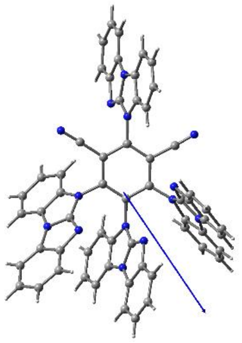<br>13.6D
(opt)	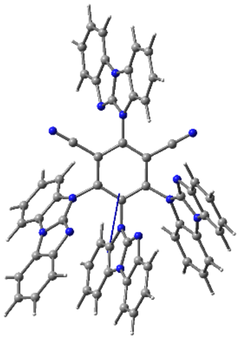<br>6.4D	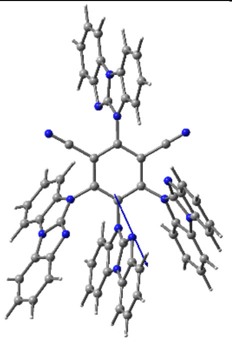<br>7.2D

Table S3. Vertical excitations $S_0 \rightarrow S_n$ and $S_0 \rightarrow T_n$, character and oscillator strength coefficients for $S_0 \rightarrow S_n$ in crystal and optimized S_0 geometry.

	Excitation energy	Character	Oscillator strength
(crystal)	S_1: 3.11eV = 399nm	HOMO – LUMO (92%)	0.0773
	S_2: 3.28eV = 378nm	HOMO-1 – LUMO (83%)	0.0341
	S_3: 3.42eV = 363nm	HOMO-2 – LUMO (59%)	0.0131
	T_1: 2.94eV = 422nm	HOMO – LUMO (71%)	
	T_2: 3.09eV = 402nm	HOMO-2 – LUMO (19%)<br>HOMO-1 – LUMO (19%)	
	T_3: 3.13eV = 396nm	HOMO-2 – LUMO (47%)	
(opt)	S_1: 3.19eV = 388nm	HOMO – LUMO (78%)	0.0906
	S_2: 3.28eV = 378nm	HOMO-1 – LUMO (78%)	0.0190
	S_3: 3.42eV = 363nm	HOMO-2 – LUMO (81%)	0.0408
	T_1: 2.99eV = 415nm	HOMO – LUMO (46%)<br>HOMO-1 – LUMO (18%)	
	T_2: 3.05eV = 406nm	HOMO – LUMO (35%)<br>HOMO-3 – LUMO (19%)<br>HOMO – LUMO+1 (16%)	
	T_3: 3.18eV = 389nm	HOMO-1 – LUMO (50%)<br>HOMO – LUMO+1 (31%)	

Table S4. HONTO and LUNTO molecular orbitals of the excited states in crystal and optimized S_0 geometry.

		HONTO	LUNTO
(crystal)	S_1	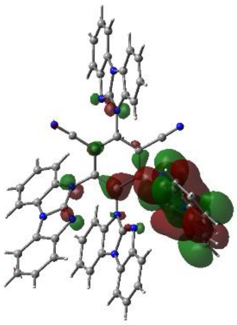	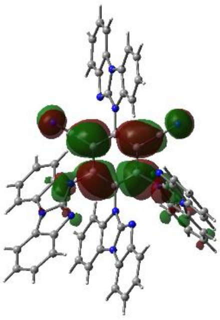
	S_2	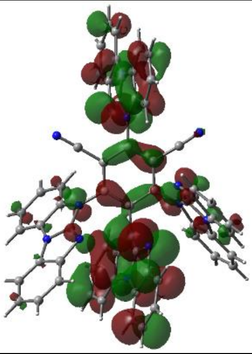	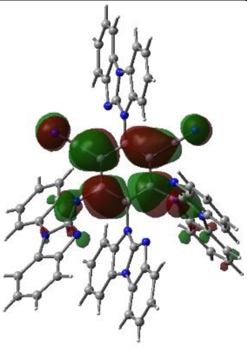
	S_3	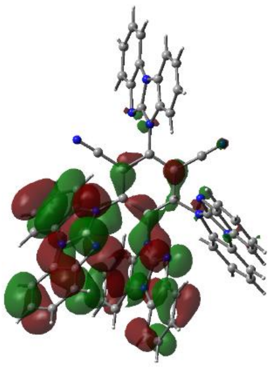	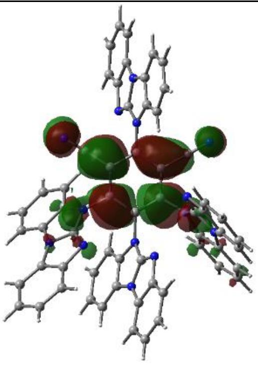
	T_1	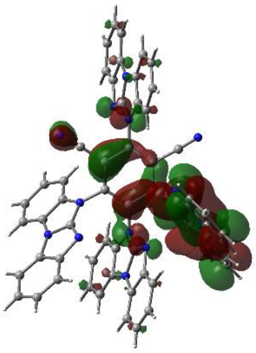	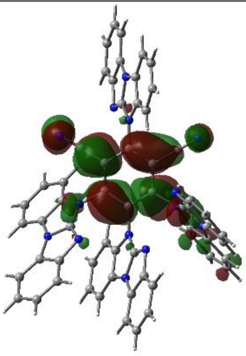

	T_2	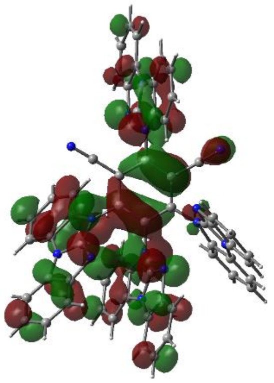	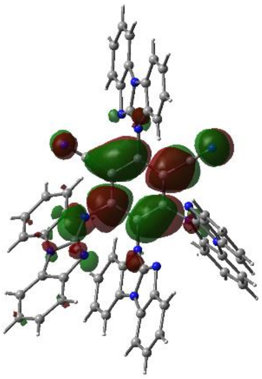
	T_3	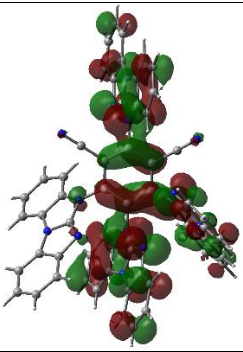	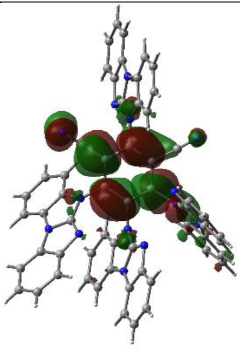
(opt)	S_1	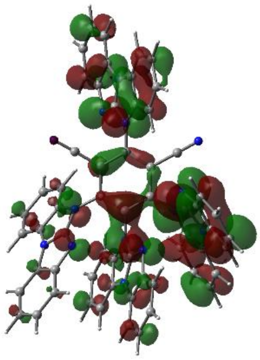	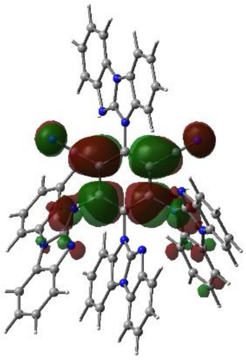
	S_2	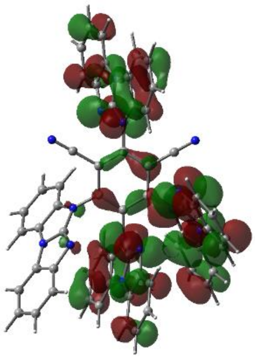	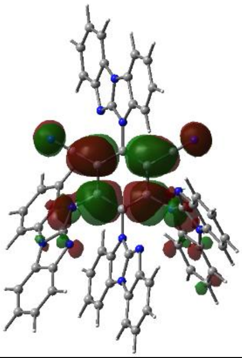
	S_3	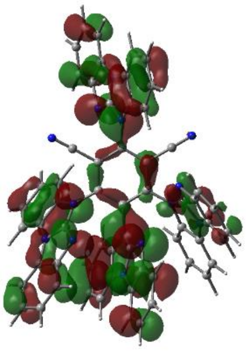	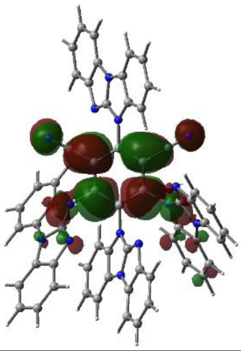

	T_1	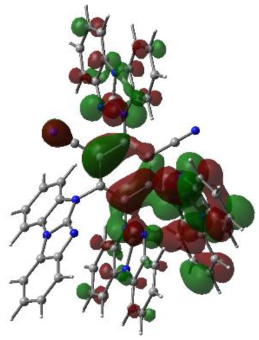	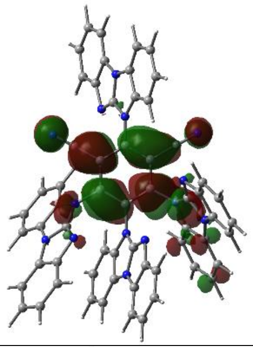
	T_2	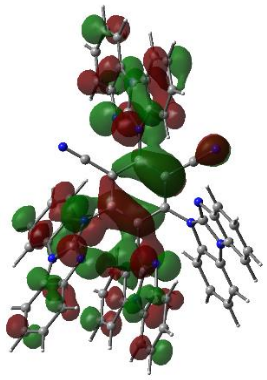	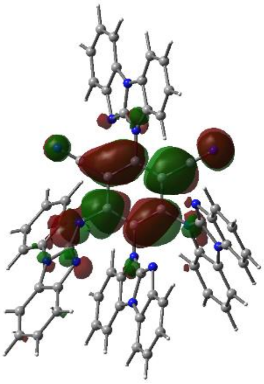
	T_3	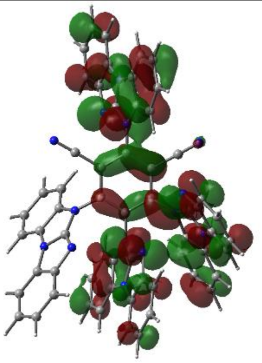	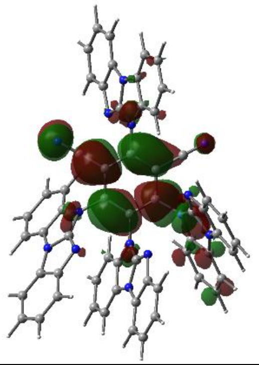

Coordinates for compound 4BGIPN in the ground state S_0 geometry (crystal geometry).

N	14.31500000	9.19000000	7.14100000
N	10.55200000	13.38100000	7.44200000
N	9.72700000	10.69700000	6.79700000
N	7.74300000	9.98600000	7.53600000
N	9.48500000	9.18400000	8.75300000
N	8.82600000	12.44200000	8.95700000
N	13.24800000	13.72600000	8.36300000
N	8.97500000	14.65100000	8.38400000
N	11.54000000	14.19500000	10.08800000
N	15.93700000	10.16600000	5.55500000
N	16.09200000	8.04800000	6.42700000
N	13.14800000	15.68000000	9.45200000
C	12.01500000	10.00100000	6.90600000
C	9.07200000	9.90900000	7.74300000
C	11.49300000	12.32100000	7.39700000
N	16.02600000	11.63900000	8.94600000
C	7.15400000	9.27800000	8.56500000

C	11.08200000	11.03100000	7.01200000
C	12.82500000	12.50700000	7.81300000
C	8.74500000	11.32800000	6.02600000
N	11.31800000	7.70900000	5.92100000
C	9.45200000	13.37800000	8.28800000
C	12.56200000	14.45100000	9.32700000
C	13.35100000	10.20600000	7.26800000
C	15.01900000	11.56300000	8.37900000
C	8.24900000	8.78500000	9.30700000
C	13.73100000	11.46500000	7.76200000
C	11.60400000	8.73400000	6.34900000
C	15.43700000	9.28700000	6.34200000
C	7.49200000	10.84800000	6.48100000
C	14.26900000	14.57500000	7.86000000
C	10.76800000	14.72000000	7.04200000
C	7.82000000	13.17900000	9.59500000
C	14.28400000	7.90200000	7.71400000
C	9.81600000	15.52700000	7.66900000
C	6.83300000	12.72200000	10.44800000
H	6.75900000	11.80200000	10.67300000
C	8.02300000	7.99700000	10.42100000
H	8.74900000	7.65100000	10.92700000
C	7.89100000	14.55400000	9.26500000
C	8.84000000	12.17900000	4.93900000
H	9.67800000	12.51600000	4.64300000
C	11.70800000	15.27000000	6.19400000
H	12.34200000	14.72800000	5.73800000
C	15.37400000	7.20800000	7.27000000
C	5.66100000	8.20200000	10.03500000
H	4.77700000	7.98400000	10.30800000
C	11.39700000	15.40800000	10.79100000
C	14.21200000	15.80400000	8.52200000
C	6.33500000	11.19100000	5.82400000
H	5.48900000	10.86900000	6.11500000
C	17.04200000	9.45900000	5.01400000
C	6.44300000	12.03000000	4.71300000
H	5.65700000	12.26300000	4.23300000
C	15.18900000	14.36900000	6.82800000
H	15.25300000	13.54100000	6.36600000
C	5.94800000	13.66700000	10.96200000
H	5.27300000	13.38000000	11.56600000
C	7.03500000	15.49100000	9.77600000
H	7.11900000	16.41600000	9.57300000
C	5.84200000	8.98400000	8.91200000
H	5.10700000	9.30400000	8.40300000
C	6.73000000	7.72600000	10.77700000
H	6.56100000	7.20000000	11.55000000
C	17.15000000	8.16300000	5.53400000
C	6.01300000	14.99400000	10.62700000
H	5.36300000	15.59400000	10.97400000

C	9.78200000	16.87200000	7.50600000
H	9.12600000	17.40500000	7.93900000
C	7.64200000	12.52100000	4.29800000
H	7.66800000	13.11100000	3.55400000
C	10.46100000	15.76700000	11.74600000
H	9.78000000	15.16100000	12.01200000
C	15.63300000	5.86600000	7.66900000
H	16.37400000	5.36100000	7.35600000
C	12.39600000	16.34100000	10.41600000
C	11.68500000	16.64200000	6.03800000
H	12.33000000	17.05600000	5.47800000
C	10.74100000	17.43200000	6.68300000
H	10.75400000	18.37400000	6.55700000
C	17.97100000	9.91300000	4.07800000
H	17.93800000	10.79500000	3.72300000
C	10.54300000	17.01500000	12.29700000
H	9.91800000	17.26000000	12.96700000
C	11.50000000	17.93100000	11.91500000
H	11.50000000	18.79900000	12.29800000
C	16.00200000	15.45300000	6.52400000
H	16.62900000	15.35800000	5.81600000
C	13.38000000	7.35200000	8.59900000
H	12.61700000	7.83300000	8.89900000
C	15.05200000	16.84200000	8.24400000
H	15.03200000	17.65400000	8.73600000
C	12.48100000	17.58700000	10.95800000
H	13.16600000	18.19500000	10.70600000
C	19.02100000	7.69900000	4.24000000
H	19.71000000	7.11400000	3.94400000
C	18.14500000	7.25200000	5.17000000
H	18.20300000	6.38300000	5.54700000
C	13.64700000	6.03800000	9.03500000
H	13.07500000	5.62500000	9.66900000
C	18.95600000	8.99100000	3.69600000
H	19.60000000	9.24900000	3.04600000
C	15.95000000	16.63600000	7.18100000
H	16.53200000	17.34100000	6.91800000
C	14.69900000	5.37300000	8.56000000
H	14.81900000	4.48000000	8.86200000

Coordinates for compound 4BGIPN in the ground state S_0 geometry (optimized geometry).

N	3.83442600	0.90870300	-0.29780500
N	-1.50230400	-0.52172100	0.65031500
N	-0.54057900	2.03640200	1.38865100
N	-2.02326100	3.70084800	1.16456000
N	-0.36054800	3.76416300	-0.37286600
N	-2.68040900	1.27132700	-0.57465100
N	0.28696900	-2.33182700	-0.65743800
N	-3.70099500	-0.56708400	0.26534200

N	-1.85021900	-1.91604700	-1.84132600
N	5.04490800	-1.00589500	0.69914400
N	6.05642500	0.74200300	-0.32996600
N	-1.16351300	-3.95898100	-1.15101700
C	1.64414000	1.43409500	0.60414200
C	-0.85532500	3.18418900	0.66862000
C	-0.15923300	-0.18673000	0.43780100
N	3.43521500	-2.11770300	-2.11996000
C	-2.37082900	4.72234800	0.29119800
C	0.30649200	1.07789400	0.81955000
C	0.71112600	-1.06550000	-0.22705000
C	-1.57814100	1.84042000	2.32279800
N	2.58001900	3.58120700	1.69188600
C	-2.55406200	0.15899500	0.06996500
C	-0.92244800	-2.61304700	-1.26797800
C	2.50160100	0.56563000	-0.07060500
C	2.82188800	-1.48119100	-1.38120600
C	-1.31154600	4.74904300	-0.64703000
C	2.02334500	-0.67187100	-0.51165900
C	2.15008200	2.64010800	1.18556800
C	4.90542300	0.11638500	0.06799500
C	-2.51542900	2.87437400	2.16721500
C	0.80584600	-3.55752900	-0.17141500
C	-2.02293100	-1.69785500	1.21251600
C	-4.04594900	1.30792700	-0.85426900
C	4.34873700	2.05799600	-0.93584100
C	-3.40397500	-1.74127100	0.94481300
C	-4.77766300	2.28639400	-1.51968800
H	-4.28099200	3.17104000	-1.90090000
C	-1.34667300	5.67694200	-1.68227800
H	-0.54657300	5.71179100	-2.40976500
C	-4.70974200	0.16327100	-0.35022500
C	-1.74900400	0.85408600	3.27227000
H	-1.01696600	0.06545600	3.39977800
C	-1.40068900	-2.69523800	1.93595500
H	-0.34290100	-2.64702800	2.16784500
C	5.74846200	1.94854800	-0.95596100
C	-3.47440100	6.48812100	-0.82268200
H	-4.30699700	7.17295000	-0.91827500
C	-2.84145800	-2.86517100	-2.08329300
C	-0.11428400	-4.57284400	-0.47623800
C	-3.66516200	2.91903100	2.93185000
H	-4.39282100	3.70876500	2.80375000
C	6.43197700	-1.16250600	0.72938900
C	-3.85405200	1.90316500	3.86694300
H	-4.74852100	1.90559800	4.47556700
C	1.95256600	-3.81980800	0.55299200
H	2.67051400	-3.04482900	0.79758900
C	-6.14178500	2.08549400	-1.67675300
H	-6.73092100	2.83081900	-2.19587400

C	-6.07028900	-0.04579400	-0.50869200
H	-6.56019900	-0.93267600	-0.12739000
C	-3.45718800	5.57950900	0.22685500
H	-4.26026700	5.53953400	0.95123300
C	-2.43441000	6.53496500	-1.75811600
H	-2.48306900	7.25915300	-2.56143500
C	7.09092800	-0.08123700	0.09661200
C	-6.77779200	0.93882000	-1.18598800
H	-7.84292200	0.81759900	-1.33548100
C	-4.17442200	-2.81904800	1.33460000
H	-5.22947300	-2.86684900	1.09996900
C	-2.91152700	0.89301700	4.03943300
H	-3.08093500	0.12361300	4.78091700
C	-4.11925600	-2.68100800	-2.60269000
H	-4.44071300	-1.69712200	-2.92147900
C	6.53472100	2.92316200	-1.53872500
H	7.61283300	2.83884500	-1.55598600
C	-2.44164700	-4.15365800	-1.65636700
C	-2.17937600	-3.78294600	2.33178700
H	-1.71384700	-4.59044600	2.88183600
C	-3.53571800	-3.84841000	2.02545300
H	-4.11048100	-4.71085200	2.33595600
C	7.19040400	-2.19087900	1.27865100
H	6.70389500	-3.02672900	1.76386700
C	-4.95699500	-3.78448400	-2.67744200
H	-5.95695300	-3.66472400	-3.07483800
C	-4.54038000	-5.05383700	-2.25640400
H	-5.22366100	-5.88954300	-2.33481300
C	2.16204900	-5.13799500	0.95563800
H	3.05622300	-5.37780900	1.51509700
C	3.69794300	3.13349400	-1.50719300
H	2.61715600	3.22133900	-1.49168400
C	0.09538300	-5.87842000	-0.07758800
H	-0.61696000	-6.65751600	-0.31310600
C	-3.26855500	-5.26245400	-1.74058700
H	-2.94901700	-6.24152600	-1.40817600
C	9.20359900	-1.03078100	0.55713800
H	10.28418500	-1.00477400	0.50420000
C	8.47016500	0.00708900	-0.00022300
H	8.95627400	0.84241300	-0.48709400
C	4.49353900	4.11538500	-2.09254100
H	4.01753300	4.97679600	-2.54113600
C	8.57162100	-2.11033500	1.18471300
H	9.17877200	-2.90136800	1.60619100
C	1.25534200	-6.14726000	0.64499200
H	1.45441100	-7.16028200	0.96864400
C	5.88207800	4.01458000	-2.10595700
H	6.46771600	4.79837700	-2.56766800
